# Emerging Roles for Intersectin (ITSN) in Regulating Signaling and Disease Pathways

**DOI:** 10.3390/ijms14047829

**Published:** 2013-04-10

**Authors:** Michael P. Hunter, Angela Russo, John P. O’Bryan

**Affiliations:** Department of Pharmacology, University of Illinois Cancer Center, Center for Cardiovascular Research, University of Illinois at Chicago, Chicago, IL 60612, USA; E-Mails: mphunter4444@yahoo.com (M.P.H.); angiusso@gmail.com (A.R.)

**Keywords:** Intersectin, endocytosis, cell signaling, Ras, PI3KC2β, Down Syndrome, Alzheimer Disease, cancer, neuroblastoma, glioblastoma

## Abstract

Intersectins (ITSNs) represent a family of multi-domain adaptor proteins that regulate endocytosis and cell signaling. *ITSN* genes are highly conserved and present in all metazoan genomes examined thus far. Lower eukaryotes have only one *ITSN* gene, whereas higher eukaryotes have two *ITSN* genes. ITSN was first identified as an endocytic scaffold protein, and numerous studies reveal a conserved role for ITSN in endocytosis. Subsequently, ITSNs were found to regulate multiple signaling pathways including receptor tyrosine kinases (RTKs), GTPases, and phosphatidylinositol 3-kinase Class 2beta (PI3KC2β). ITSN has also been implicated in diseases such as Down Syndrome (DS), Alzheimer Disease (AD), and other neurodegenerative disorders. This review summarizes the evolutionary conservation of ITSN, the latest research on the role of ITSN in endocytosis, the emerging roles of ITSN in regulating cell signaling pathways, and the involvement of ITSN in human diseases such as DS, AD, and cancer.

## 1. Introduction

**Figure 1:**
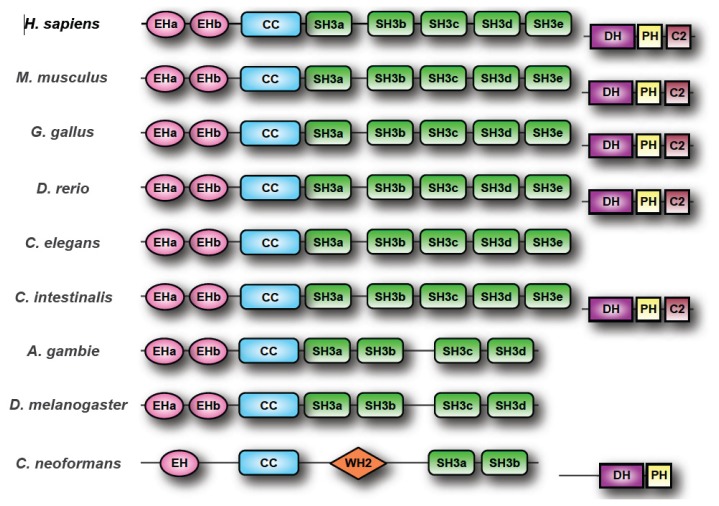
ITSN structure. ITSN proteins are comprised of Eps15 homology (EH) domains, a coiled-coil (CC) domain, and multiple Src homology 3 (SH3) domains, each with distinct ligands. In many organisms, a longer splice product is also present. This longer product, termed ITSN-L, shares all the domains with the shorter splice product, referred to as ITSN-S, but in addition possesses a *C*-terminal extension encoding a Rho exchange factor domain (DH) that functions in concert with the PH domain to activate Cdc42. ITSN orthologs have been found in the genomes of all metazoans to date (Table 1) although the DH–PH–C2 region is not conserved in all ITSN orthologs. Most ITSNs possess 2 EH domains and 5 SH3 domains although a number of lower eukaryotic ITSNs have fewer SH3 domains. For example, *C. neoformans* ITSN (Cin1) has a single EH domain and 2 SH3 domains and no C2 domain in ITSN-L. In addition, Cin1 has an added WH2 domain, which is involved in binding actin.

Intersectin (ITSN) is a multi-domain, scaffolding protein first identified in *Xenopus*[1,2]. ITSN orthologs have since been identified in all metazoan genomes examined thus far. *ITSN* genes encode two main protein isoforms that arise from differential splicing (Figure 1). The short isoform (ITSN-S) contains two *N*-terminal Eps15 homology (EH1 and EH2) domains, a coiled coil (CC) region, and five Src homology 3 (SH3A-SH3E) domains. ITSN-S isoforms are expressed ubiquitously, with the exception of neurons [3–7]. The long isoform (ITSN-L) is co-linear with ITSN-S, but in addition has an extended *C*-terminus encoding a Dbl homology (DH) domain, a pleckstrin homology (PH) domain, and a C2 domain. Mammals have two ITSN-L isoforms, ITSN1-L and ITSN2-L. ITSN1-L expression is specific to the brain, whereas ITSN2-L expression is ubiquitous [8]. The presence of the EH domains suggested that ITSN has a role in endocytosis. In support of this, multiple studies demonstrated that the EH domains directly interact with components of the endocytic machinery such as Epsin 1 and 2, Rev interacting protein (Rip/RAB/Hrb), SCAMP1, and Stonin2 [2,9–11]. Also, ITSN localizes to clathrin-coated vesicles, and regulates clathrin-dependent endocytosis [7,12]. Further, ITSN plays a conserved role in endocytosis and vesicle recycling in multiple species (reviewed in [13]). Although ITSNs have a well-established role in endocytosis, the exact mechanisms by which ITSNs function are still being defined. This review will discuss recent findings into how ITSN scaffolds function in regulating endocytosis. In addition to its role in endocytosis, ITSN has emerged as a key regulator in a growing list of cell signaling pathways including the epidermal growth factor receptor (EGFR), Ras, and PI3KC2β. This review will discuss recent findings on ITSN’s role in regulating these signal transduction pathways and insights into additional pathways regulated by these scaffolds. Finally, this review will highlight recent findings on ITSN’s role in a number of pathological conditions including neurodegeneration and cancer

## 2. The *ITSN* Gene Family

### 2.1. *ITSN* Orthologs

*ITSN* orthologs are found in all complete metazoan genomes examined thus far (Table 1). Lower organisms typically have a single *ITSN* gene. Mammals have two *ITSN* genes (*ITSN1* and *ITSN2*), each encoding both a short (ITSN-S) and a long isoforms (ITSN-L) due to differential splicing of each gene (reviewed in [13]) (Figure 1). The DH-PH domains encoded by the extended *C*-terminus of ITSN-L isoforms function in concert as a guanine nucleotide exchange factor (GEF) specifically for Cdc42 [14–17]. Several interesting observations arise from comparison of these ITSN orthologs. First, the majority of these proteins possess domain structures identical to human ITSN proteins with a few notable exceptions: several insect ITSN orthologs possess only 4 SH3 domains while most other ITSN proteins have the characteristic 5 SH3s. Second, several bony fish genomes, with the exception being *Oryzias latipes*, appear to have undergone genome duplication resulting in the presence of three *ITSN* genes. Third, ITSN orthologs in higher eukaryotes have the DH–PH modules to allow for regulation of Cdc42, whereas ITSN orthologs in many lower eukaryotes lack these modules. This observation suggests that the primordial functions of ITSN lie in the shorter isoform. However, an ITSN1 ortholog, Cin1 has been described in the pathogenic fungus *Cryptococus neoformans*[18]. *Cin1* encodes both short and long isoforms. Cin1-L (long isoform) has a domain organization similar to ITSN-L isoforms in higher eukaryotes, with the exception that Cin1-L has a single *N*-terminal EH domain, a coiled coil region, two SH3 domains, and *C*-terminal DH–PH domains [18]. Conservation of the GEF domain in Cin1 suggests that the long isoform of ITSN arose at a much earlier time in the evolution of this gene family, and that regulation of both endocytosis and the actin cytoskeleton were primordial functions of ITSN proteins. Perhaps both short and long isoforms of ITSNs were present during early evolution of the ITSN protein family. However, this possibility raises the additional question of why the DH–PH domain of ITSN-L is conserved in Cin1, but not other lower eukaryotes? Shen and colleagues [18] demonstrate that *cin1* mutants lacking the GEF domain, or the GEF and SH3 domains, did not exhibit any phenotype. The authors concluded that these domains were dispensable for Cin1 function, and that other functionally redundant proteins compensate for the lack of these domains. It is possible that the DH–PH domains of ITSN orthologs in the lower eukaryotes were lost during evolution of these genes. Perhaps GEF function was compensated for by other proteins that interact with these ITSN orthologs. In addition to the aforementioned domains, Cin1 possesses a central WASP homology 2 (WH2) domain for binding to monomeric actin. The presence of these domains in Cin1 may further suggest a primordial role for ITSN in regulating actin dynamics. Surprisingly, proteins homologous to Cin1 are found in other basidiomycetus fungi, but absent in zygomycetus fungi and ascomycetus fungi such as *S. cerevisiae*[18]. Further examination of the sequences of *ITSN* orthologs from lower eukaryotes may help to reveal when the GEF domain was lost in some groups.

### 2.2. ITSN Splice Variants and Altered Functions

In addition to the major splice variants of ITSN (*ITSN-S* and *ITSN-L*), there are a number of minor splice variants that occur throughout the protein resulting in changes in the EH, SH3, and DH domains (reviewed in [13,21]). The SH3 domains of ITSNs regulate protein–protein interactions with targets such as dynamin, SOS, Cbl, and WASP [2,12,22,23]. Thus, splicing variants in these regions may affect how ITSNs interact with their binding partners. One study examined the biochemical consequences of splicing a neuron-specific exon (microexon 20) within the SH3A domain of ITSN1, resulting in the inclusion of 5 additional amino acids in the n-Src loop of SH3A [24]. These additional amino acids reduced binding to SOS and Cbl, compared to ITSN1-S. Dergai and colleagues showed that microexon 20 in the SH3A domain of ITSN1 regulates interaction with dynamin 1 [25]. The authors examined the genomic sequences of *ITSN1* from human, mouse, rat, chicken, and zebrafish, and found that microexon 20 was conserved. Microexon 20 transcripts were also expressed in zebrafish at the beginning of neurogenesis, and were specific to the brain. This microexon causes a shift of negative amino acids towards the interaction interface of the SH3A domain with the Pro-rich domain (PRD) of dynamin 1. Dynamin 1 bound more efficiently to the neuron-specific form of the SH3A (N-SH3A) than the ubiquitously expressed form of the SH3A. The authors concluded that microexons represent a conserved method of controlling the tissue specific interactions of ITSN1 with its binding partners.

Additional splicing events have been reported including one that results in truncation of exon 6 and deletion of 37 amino acids between the EH domains. Another splicing event involving exons 25 and 26 results in an isoform lacking SH3C [6,21]. Further, minor splice variants of ITSN1 and ITSN2 have been reported that result in truncated versions of these proteins containing only the EH and CC domains (reviewed in [13,21]). Additionally, Kropyvko and colleagues used RT-PCR on human brain and lung tissue to identify 12 novel transcriptional isoforms of *ITSN1-S* and 3 novel isoforms of *ITSN1-L* containing full-length coding sequences [26]. The authors demonstrated that these novel isoforms differ in domain organization, expression levels, and tissue patterns of expression. For example, alternative splicing of exon 35 in *ITSN1-L* resulted in an isoform lacking 31 AA’s from the DH domain and 25 AA’s from the region between the DH and PH domains. The transcripts containing exon 35 were expressed at higher levels than transcripts lacking exon 35. Also, transcripts with and without exon 35 were predominantly detected in the brain. The identification of novel isoforms of ITSN adds to the complexity in the regulation of ITSN function in different tissues.

## 3. The Role of ITSN in Endocytosis: New Insights

The functional role of ITSN in endocytosis and intracellular trafficking are conserved in lower organisms. Shen and colleagues [18] demonstrated that *Cin1* null mutants had defects in cell morphology, failed to divide properly, and had abnormal distribution of actin. Additionally, *cin1* mutants showed defective endocytosis as demonstrated by a failure in uptake of a vacuolar membrane dye. Finally, a yeast two-hybrid assay was used to show that the Cin1 DH domain interacts with cryptoccocal Cdc42, and the SH3 domains interact with the cryptococcal homologue of WASP (Wsp1). The fact that Cin1 interacts with Cdc42 and Wsp1, similar to the interaction of ITSN1-L with Cdc42 and WASP, suggests a conserved role in regulation of the actin cytoskeleton. To test whether Cin1, Cdc42, and Wsp1 function in the same pathway, Shen and colleagues examined epistatic relationships among these proteins [27]. Expression of a constitutively active Wsp1 mutant partially rescued the endocytic defect in *cin1* mutants. A constitutively active *Cdc42* mutant rescued the defect in actin organization in the *Cin1* mutant. Further, expression of the Cin1 DH domain rescued the actin defect in *wsp1* mutants. The authors concluded that Cin1 functions in intracellular trafficking and regulation of the actin cytoskeleton through Cdc42 and Wsp1, similar to ITSN1 in mammals. In *C. elegans*, ITSN-1 localizes to sites of synaptic vesicle recycling and serves to recruit components of the endocytic machinery [28,29]. Loss-of-function mutants of ITSN-1 in *C. elegans* resulted in decreased numbers of synaptic vesicles and reduced synaptic vesicle recycling [28,29]. The *Drosophila* ortholog of human ITSN1, dynamin associated protein of 160 kD (Dap160), was isolated based on its association with the GTPase dynamin [30]. Dap160 serves as a scaffold for endocytic proteins, and functions in vesicle endocytosis and recycling at the neuromuscular junction [31,32]. In *D. melanogaster*, Dap160 controls synaptic development, and deletion of Dap160 is embryonic lethal. Dap160 loss-of-function mutants have decreased synaptic vesicle recycling, and form abnormally large vesicles.

ITSN also plays an important role in endocytosis and vesicle trafficking in mammals. Mice lacking ITSN1 have vesicle trafficking defects in neurons in the brain [3]. Silencing of ITSN1 in human embryonic kidney (293T) cells decreases uptake of EGFR [23]. Knockdown of *ITSN1* using either shRNAs that target both *ITSN1-S* and *ITSN1-L* or shRNAs specific to *ITSN1-L* reduces internalization of the transferrin receptor in hippocampal neurons [33]. In endothelial cells, silencing *ITSN1-S* reduces caveolae-dependent endocytosis [34,35]. Overexpression of ITSN, in multiple cell types, inhibits endocytosis (reviewed in [13,21]). The fact that both silencing and overexpression of ITSN result in endocytic defects most likely is due to a concentration-dependent effect of this scaffold protein in the proper regulation of endocytosis (reviewed in [13]). Thus, regulation of ITSN levels will have important biochemical consequences to cell function.

ITSNs directly interact with components of the endocytic machinery. Dap160, binds the endocytic protein dynamin [30] which regulates endocytic membrane fission. The SH3 domains of ITSN1-S bind dynamin-1, which is predominantly expressed in the brain, and dynamin-2, which is ubiquitously expressed [36,37]. Further, dynamin and ITSN1-S are required for caveolae-mediated endocytosis [35,38]. The SH3A domain of ITSN1 displays the highest affinity for dynamin-2, and it is a potent inhibitor of endocytosis [2]. How the SH3A domain interferes with dynamin-2-mediated endocytosis is not completely understood. Knezevic and colleagues overexpressed the SH3A domain in cultured human lung endothelial cells (EC’s) to examine its effects on endocytosis [39]. SH3A overexpression disrupted caveolae internalization in EC’s. Additionally, SH3A domain overexpression stimulated dynamin-2 assembly and stabilized dynamin-2 oligomers. The authors concluded that SH3A regulates dynamin-2 assembly and disassembly in the process of caveolae-dependent endocytosis. Further, Predescu and colleagues tested the effect of overexpression of the SH3A domain *in vivo*[40]. The authors modulated ITSN1-S function in mouse lung endothelium by overexpression of ITSN1 SH3A domain, or by siRNA to silence *ITSN1* expression. These studies demonstrated that ITSN1 is required for caveolae function in transendothelial transport. The expression of SH3A stabilized dynamin-2 oligomers and impaired dynamin-2 function such that caveolae could not detach from the plasma membrane. This *in vivo* effect is similar to that seen with SH3A overexpression in EC’s in cell culture [39]. Furthermore, acute depletion of ITSN1-S resulted in increased endothelial permeability and impaired interendothelial junctions [40]. Interestingly, chronic depletion of ITSN1-S resulted in the upregulation of alternative endocytic pathways. These studies provide new insights into how the interaction of dynamin and the ITSN1 SH3A domain regulates endocytosis.

ITSN1 interacts with additional components of the endocytic machinery such as the heterotetrameric clathrin adaptor complex AP2. Injection of antibodies directed against the SH3 domains of ITSN1 into lamprey synapses inhibited endocytosis [41]. To further examine the mechanism underlying this inhibition of endocytosis, Pechstein and colleagues developed specific antibodies to each ITSN1 SH3 domain [42]. Microinjection of a specific antibody to the SH3A-B linker region inhibited synaptic vesicle (SV) recycling in lamprey synapses. Immunoprecipitation studies demonstrated that ITSN1 associated with the clathrin adaptor AP2 through interaction of the ITSN1 SH3A-B linker region with the AP2 and appendage domains. Further, binding of AP2 to ITSN1 inhibited the interaction of ITSN1 with the endocytic protein synaptojanin 1. The authors concluded that the SH3A-B linker region of ITSN1 plays an important role in early stages of SV recycling, and that the ITSN1-AP2 complex is an important regulator of SV recycling at synapses.

The interaction network of ITSN proteins in endocytosis continues to expand. Dergai and colleagues investigated the interaction of ITSN1-S with other proteins of the endocytic machinery [43]. An *in silico* based approach identified the membrane deforming protein SGIP1 [Src homology-3 domain growth factor receptor bound 2-like (endophilin) interacting protein 1] and the adaptor protein Reps1 (RalBP associated Eps-15 homology domain protein) as potential ITSN1 binding partners. Immunoprecipitation and *in vitro* binding experiments showed that ITSN1-S and SGIP1 interact, and that this interaction involved the SH3A and SH3E domains of ITSN1-S, and the PRD of SGIP1. ITSN1-S and SGIP1 co-localized to the plasma membrane in discrete spots that were also clathrin positive. Similar experiments revealed that ITSN1-S interacted with Reps1, and that this interaction involved the SH3C domain of ITSN1-S and the PRD domain of Reps1. Further, ITSN1-S and Reps1 also co-localized to the plasma membrane in clathrin-coated pits. Thus, SGIP1 and Reps1 bind independent sites on ITSN1. Immunoprecipitation experiments demonstrated that ITSN1-S, SGIP1, and Reps1 are present in the same molecular complex. The authors concluded that ITSN1 functions as a platform to recruit both SGIP1 and Reps1 These results further extend the interaction network between adaptor proteins and membrane deforming proteins involved in clathrin-mediated endocytosis [13,44].

## 4. A Growing Number of ITSN-Regulated Cell Signaling Pathways

### 4.1. Identification of Novel ITSN Binding Partners by Yeast Two-Hybrid Screening

Although ITSN1 was initially identified as an endocytic scaffold protein, subsequent studies have revealed that ITSN1 regulates cell signaling pathways including multiple Ras family GTPases (including Ras, Rac, and Cdc42), EGFR, PI3KC2β, JNK, WNKs, aPKC, and SHIP2 (reviewed in [13,21]). However, the mechanisms by which ITSNs regulate these pathways are not fully understood. In an attempt to identify ITSN targets important in regulating these aforementioned pathways as well as identify new ITSN-regulated pathways, Wong and colleagues performed a high-throughput yeast two-hybrid screen with ITSN1 and ITSN2 [45]. More than one hundred new ITSN targets were identified, including 55 binding partners for ITSN1, 62 binding proteins for ITSN2, and 10 binding partners common to both. Many of these ITSN-binding proteins (21) are involved in endocytosis including previously identified ITSN targets such as clathrin, dynamin 2, and Eps15, as well as novel ITSN-binding partners such as KIF16B, syntaxin 4, and the clathrin assembly lymphoid myeloid leukemia protein (CALM). ITSN1 was also isolated as a binding partner for itself. Indeed, ITSN1 and ITSN2 homodimerize and heterodimerize [45], which adds a new level of complexity to ITSN-regulated pathways. Given the ability of ITSN to heterodimerize with Eps15 [7], coupled with the multiple isoforms of ITSNs as well as Eps15-related proteins, these findings suggest the existence of a wide number of complexes between these multi-domain scaffolding proteins, each of which may associate with a specific array of targets. Therefore, a future challenge will be to determine which of these complexes are biochemically and physiologically relevant.

A role for ITSN in the regulation of microtubule dynamics is beginning to emerge. ITSN1 associates with the microtubule stabilizing protein Stable Tubule Only Polypeptide (STOP) in primary hippocampal neurons [46]. In addition, ITSN1 binds DISC1 (deleted-in-schizophrenia 1), which is a centrosome-associated protein that regulates synaptic vesicle transport along microtubules [47]. Several additional ITSN binding proteins are also associated with microtubule dynamics including TACC1, clathrin (through binding of TACC3), PDE4D-interacting protein (PDE4DIP/myomegalin), and CLIP-115 [45]. These interactions suggest that ITSN may coordinate the association of cargo with microtubules to facilitate transport. Indeed, loss of function of ITSN1 leads to defects in vesicle transport and recycling in a number of cellular contexts (reviewed in [13]). These associations also suggest a potential role of ITSN1 in psychiatric disease as well (see below).

### 4.2. ITSN Regulates Ras Superfamily Members

Ras family proteins regulate many processes in the cell including proliferation, migration, and MAPK signaling. Ras GTPases cycle between an active GTP-bound form and an inactive GDP-bound form. This cycling is regulated by GEFs that promote release of GDP and subsequent binding of GTP to activate Ras. GTPase activating or accelerating proteins (GAPs) enhance the intrinsic GTPase activity to promote GTP hydrolysis to terminate Ras GTPase activity. ITSN1 regulates activation of several Ras family GTPases including H-Ras, Cdc42, and Rac1 [13]. Additionally, multiple regulators of Ras superfamily members have been identified as ITSN-interacting proteins, including GEFs such as Sos, TRIO, P-Rex, cytohesin 1, and TIAM1, and GAPs such as CdGAP, the p85 subunit of PI3K, and HRB/HRB-L [45,48]. Furthermore, several effectors of Arf and Rab GTPases were identified as ITSN-binding proteins supporting a role for ITSNs in regulating vesicle trafficking [45]. Thus, ITSNs likely serve as a nexus for regulation of multiple Ras family GTPase pathways (see below).

ITSN-L isoforms regulate Cdc42 activity through their C-terminal DH and PH domains which function as Cdc42-specific GEFs [14–16]. Over-expression of the ITSN1-L GEF domain activated Cdc42 signaling in fibroblasts [15], and the presence of the SH3 domains inhibited GEF activity [16] suggesting an auto-inhibited conformation of ITSN1-L between the SH3s and GEF domains. To more closely examine this mechanism of auto-inhibition, Ahmad and Lim [49] used deletion mutants of ITSN1-L and crystallography for structural analysis. The authors demonstrated that the SH3 domains of ITSN1-L are important for the auto-inhibitory regulation of ITSN1-L GEF activity, with the SH3E domain playing the greatest inhibitory role. Analytical ultracentrifugation and gel filtration revealed that the SH3E domain inhibits the DH domain by an intramolecular interaction, which may disrupt the structure of the DH domain. In contrast, Kintscher and colleagues examined the mechanism of auto-inhibition of ITSN1-L GEF activity, and identified a short auto-inhibitory sequence between the SH3E and DH domains [50]. The authors analyzed nucleotide exchange rates for truncated and mutant forms of ITSN1-L. The auto-inhibition of GEF activity was due to a short linker sequence of 14 amino acids between the SH3E domain and the DH domain. A key amino acid, W1221, was essential to the auto-inhibition of GEF activity. They also examined the effect of SH3 binding of the Cdc42 effector, N-WASP, on nucleotide exchange rates. N-WASP did not disrupt auto-inhibition of ITSN1-L GEF activity, in contrast to previous results [15]. Further experiments will be required to clarify the mechanism of ITSN1-L auto-inhibition, and the role of N-WASP in regulating ITSN1-L GEF activity.

Novokhatska and colleagues examined the function of ITSN2-L in embryonic development of *Xenopus laevis*[20]. ITSN2-L transcripts were detected in the two-cell stage through the tadpole stage. Since the actin cytoskeleton plays important roles in the gastrulation movements in early development, the authors examined ITSN2-L’s role in regulating actin dynamics in early development. Microinjection of mRNA encoding the DH–PH domains into embryos resulted in hyperpigmentation and gastrulation failure. Overexpression of a constitutively active Cdc42 mutant led to gastrulation defects that resembled the phenotype observed with overexpression of the DH–PH region of ITSN2-L. Co-injection of the DH–PH domain mRNA along with mRNA encoding dominant-negative Cdc42 partially rescued the phenotype caused by the over-expression of the DH–PH domains. The authors concluded that ITSN2-L regulates Cdc42 activity and actin cytoskeleton dynamics during early *Xenopus* development. Although these results provide insight into how the DH–PH domains functions on their own, the role of these domains in *Xenopus* development will need to be examined further in the context of the whole ITSN2-L protein.

Cdc42 regulates the orientation of the mitotic spindle [51], controls cell polarity, and the formation of a single lumen during epithelial morphogenesis of MDCK cells in three dimensional (3D) cell culture [52]. Rodriguez-Fraticelli and colleagues demonstrated that ITSN2 regulates Cdc42 in cells to control mitotic spindle orientation during lumen morphogenesis in the 3D MDCK culture system [53]. Cells silenced for ITSN2 gave rise to cysts that failed to properly form a lumen, and instead formed multi-lumenal cysts. Silencing of ITSN2 resulted in a significant decrease in Cdc42-GTP levels, suggesting that Cdc42 has a role in this phenotype. Expression of a constitutively active mutant of Cdc42 (Cdc42-V12) rescued normal lumen formation in *ITSN2*-silenced cells. ITSN2 depleted cells also exhibited abnormal spindle pole orientation during cell division which was mirrored by silencing Cdc42. Interestingly, ITSN2 localized to the centrosomes. The authors concluded that ITSN2 regulates spindle pole orientation during lumen morphogenesis through activation of Cdc42 at the spindle pole. This connection of ITSN2 to the mitotic spindle is interesting given the potential connection of ITSN1 with microtubule dynamics as discussed above.

Although ITSN1-S lacks a GEF domain, it also has a role in regulation of GTPases. ITSN1 associates with the Ras GEF Sos1, and overexpression of ITSN1’s SH3 domains inhibit activation of Ras suggesting a role for ITSN1 in Ras activation [22,54]. Indeed, ITSN1-S overexpression activates Ras on perinuclear vesicles without activating the JNK or ERK MAPK pathways [55,56]. While this activation of Ras was initially postulated to be through the recruitment of GEFs, such as Sos1, to stimulate nucleotide exchange on Ras [55], a new twist on this mechanism was recently proposed. ITSN1 binds and activates a novel PI3K isoform, PI3KC2β [57]. Like Class 1 PI3Ks, PI3KC2β possesses a canonical Ras binding domain (RBD) and co-localizes with both Ras and ITSN1 on vesicles [58]. However, unlike typical Ras effectors PI3KC2β did not interact with activated Ras but rather preferentially associated with inactive Ras. Biochemical characterization of this interaction revealed that nucleotide-free Ras bound the RBD of PI3KC2β, and inhibited the *in vitro* lipid kinase activity of the enzyme. In addition, the interaction of PI3KC2β with nucleotide-free Ras prevented loading with nucleotide. Wong and colleagues propose that ITSN1 interaction with PI3KC2β results in dissociation of this nucleotide-free Ras-PI3KC2β complex resulting in immediate GTP loading onto Ras leading to its activation. The potential biological consequence of this model with respect to oncogenesis is discussed later in the review.

### 4.3. ITSN Regulation of Receptor Tyrosine Kinases (RTK): New Insights

RTK signaling pathways regulate many important biological processes in cells such as proliferation, differentiation, and apoptosis. Indeed, aberrant RTK signaling underlies a number of disease pathologies, in particular cancer. Ubiquitylation and endocytosis play important roles in regulation of RTK function. ITSN1 enhances RTK ubiquitylation through activation of the E3 ubiquitin ligase Cbl [23]. In addition, ITSN1 interacts with a number of Cbl-regulatory proteins such as Sprouty 2 (Spry2), Alix, and CIN85 [45,59,60]. Okur and colleagues demonstrated that ITSN1 binding of Spry2 interferes with Cbl-Spry2 interaction thereby disrupting the repression of Cbl by Spry2 to enhance EGFR ubiquitylation [59]. However, both Spry2 and Cbl bind the same SH3 domains in ITSN1 raising the question of how ITSN1 coordinates the interaction with each of these targets. One possibility is that ITSN dimerization provides a mechanism by which multiple SH3 binding proteins can interact with a larger ITSN complex to coordinately regulate Cbl activity. Such a multimeric complex may allow for interaction with additional regulators of Cbl, e.g., CIN85 or Alix. It will be interesting to determine how the interaction of Alix and CIN85 with ITSN1 alters the regulation of Cbl and whether there are additional components through which ITSN1 regulates Cbl function.

ITSN1 and ITSN2 bind the receptor-associated late transducer (RALT) [61]. RALT is an inhibitor of the EGFR [62,63] that blocks catalytic activation by binding the kinase domain of EGFR and inhibiting formation of the asymmetric kinase dimer [64]. However, Frosi and colleagues demonstrated that binding of RALT to EGFR affects its internalization [61]. RALT-bound EGFR undergoes internalization even when EGFR catalytic function is inhibited by mutation. Using deletion mutants of RALT, they identified an endocytic domain of RALT capable of driving EGFR endocytosis. This region binds the SH3s of both ITSN1 and ITSN2. Silencing one or both ITSNs decreased RALT-mediated endocytosis of an internalization defective mutant of EGFR. However, the effect of ITSN2 knockdown was much greater than that of ITSN1. Thus, RALT couples the EGFR to clathrin-mediated endocytosis by the interaction with ITSNs.

### 4.4. ITSNs Interact with Herpesvirus Proteins

ITSN2 interacts with the K15 protein from Karposi’s sarcoma-associated herpesvirus through the SH3C of ITSN2 binding a PPLP motif in K15 [65]. Recently, Dergai and colleagues demonstrated that ITSN1 interacts with the Latent Membrane Protein 2 (LMP2A) from Epstein-Barr virus [66]. Although the binding of LMP2A is mediated by the SH3 domains of ITSN1, SH3D appears to be the predominant domain for binding. In addition, the interaction of ITSN1 and LMP2A appears to be more complex than ITSN2-K15 interaction, involving tyrosine phosphorylation of LMP2A and recruitment of additional adaptor proteins such as Shb. These interactions promote the recruitment of the Src-like kinase Syk, which results in tyrosine phosphorylation of ITSN1. Although ITSN2 tyrosine phosphorylation has been reported in several global phosphoproteome screens, this is the first report of ITSN1 tyrosine phosphorylation. However, the role of this modification in ITSN1 function and the importance of ITSN1 to herpesvirus biology remain to be determined.

## 5. ITSN in Neurobiology: Implications for Down Syndrome (DS) and Alzheimer Disease (AD)

### 5.1. ITSN1 Regulates Dendritic Spine Morphogenesis and Survival of Neurons

ITSN1 has important roles not only in vesicle trafficking, but also in formation of neuronal cellular processes and cell survival. In *Drosophila*, loss-of-function Dap160 mutants result in defects in synaptic boutons and aberrant organization of presynapses at neuromuscular junctions [31,32]. In mammals, ITSN1-L regulates dendritic spine development of hippocampal neurons [33], along with Numb [67], and EphB receptors [68]. Silencing *ITSN1-L* in hippocampal neurons resulted in defective spine development [33]. ITSN also plays a role in neural stem cells. Dap160 activates atypical PKC (aPKC) in *Drosophila* neuroblasts and plays an important role in the proliferation of these cells [69]. Further, silencing ITSN1 in neuronal cells resulted in decreased survival of differentiating cells [57]. This phenotype was rescued by overexpression of either PI3KC2β or AKT suggesting that ITSN1 regulates an important PI3KC2β-AKT survival pathway [57].

ITSN’s association with the microtubule network provides another avenue through which this scaffold may regulate neuron biology. Transport along the microtubule network is critical for maintaining axonal connectivity and disruptions to this transport process underlie a number of neurodegenerative diseases [70]. ITSN’s interactions with proteins involved in microtubule dynamics (e.g., STOP, DISC1, PDE4DIP/myomegalin, and CLIP-115) as mentioned earlier suggest that alterations in ITSN may affect neuron function. Indeed, silencing *ITSN1* as well as genomic deletion of mouse *ITSN1* lead to synaptic vesicle recycling defects as well as exocytic defects (reviewed in [13,21]). Furthermore, deletions within HSA 21q22.11–12 region encompassing *ITSN1* lead to mental retardation [71–73]. Together, these findings suggest that ITSN1 is indeed important for normal brain function.

### 5.2. ITSN1, DS and AD

DS is characterized by trisomy of all or part of chromosome 21 (HSA21), and is the most common viable chromosomal trisomy in humans [74]. DS patients are often characterized by heart defects, musculoskeletal abnormalities, mental retardation, and early onset AD-like neuropathology [75]. Multiple lines of evidence suggest that ITSN1 has a role in both DS and AD. First, ITSN1 mRNA and protein levels are elevated in DS patient samples [76,77]. Second, ITSN1 is one of the most highly induced transcripts in AD brain samples [78–80] and the only HSA21 gene overexpressed in all three studies [78]. Third, one of the earliest phenotypic characteristics of DS and AD brains is an enlarged early endosomal compartment, suggestive of defects in endocytic trafficking [81]. Fourth, alterations in ITSN1 expression in model systems result in defects in endocytosis and receptor trafficking suggesting that the aforementioned endosomal anomalies may be due to alterations in ITSN1 expression (reviewed in [13,82]). Fifth, the ITSN1 gene is localized to a region of HSA21, 21q.22.1–q22.2, which plays a major role in the DS phenotype [83]. Finally, ITSN1 overexpression has been implicated in neurodegenerative diseases through activation of JNK [84]. These findings suggest that ITSN1 overexpression may contribute to both DS and AD. Indeed, Hunter and colleagues demonstrated that overexpression of ITSN1-S in the forebrains of mice, particularly the striatum, resulted in sex-dependent decrease in locomotor activity in the transgenic animals [77]. The level of ITSN1-S overexpression in the transgenic brains was comparable to the level of ITSN1-S observed in human DS patients. These findings suggest that overexpression of ITSN1 may contribute to phenotypes in DS individuals.

## 6. ITSN in Cancer

### 6.1. ITSN Regulates Oncogenic Signaling Pathways

ITSN1 overexpression in rodent fibroblasts promotes oncogenic transformation of cells suggesting a role for this scaffold in tumorigenesis [85]. Indeed, ITSN1 regulates a number of signaling pathways involved in tumorigenesis including RTKs and Ras (as reviewed in [13]). Several recent studies provide direct evidence for ITSN1 involvement in human cancers. Russo and O’Bryan demonstrated that ITSN1 is highly expressed in primary neuroblastoma (NB) tumors and tumor cell lines [86]. NB is the most common pediatric extracranial solid tumor. Although a fraction of these tumors spontaneously regress, many prove to be fatal. While NBs are believed to originate from neural crest, ITSN1-S is the predominant isoform expressed in NBs even though ITSN1-L is the predominant isoform in neuronal cells. Silencing ITSN1 expression reduced anchorage independent growth both *in vitro* and *in vivo*. Similar results were observed when silencing ITSN1 in glioblastoma tumor cell lines, which also appear to express predominantly ITSN1-S [87,88]. These later studies demonstrated that loss of ITSN1 resulted in decreased PAK1, LIMK1, AKT, and integrin β activation suggesting that ITSN1 regulates a migration pathway critical for tumorigenesis. In the case of NBs, overexpression of the ITSN1 target PI3KC2β rescued the anchorage-independent growth of ITSN1- silenced cells suggesting a role for this ITSN1 effector in human cancers. Indeed, PI3KC2β may contribute to tumorigenesis. PI3KC2β overexpression in colonic epithelial cells results in oncogenic transformation [89]. Single-nucleotide polymorphisms in the promoter region of PI3KC2β are associated with increased risk for prostate cancer suggesting enhanced expression of the protein in this cancer [90]. Furthermore, PI3KC2β mRNA is elevated in a variety of cancers, including pancreatic cancers [91], mixed lineage leukemias [92], and a subset of acute myeloid leukemias [93]. A recent study indicates that PI3KC2β contributes to the tumorigenic properties of acute myeloid leukemia, glioblastoma, medulloblastoma and small cell lung cancer [94]. ITSN1 is overexpressed in pancreatic cancers [95,96] suggesting that upregulation of the ITSN1-PI3KC2β signaling pathway may contribute to development of pancreatic cancers as well. Therefore, further efforts to identify the signaling pathways regulated by ITSN-PI3KC2β complex in cancer may have important therapeutic implications for multiple tumor types.

**Figure 2:**
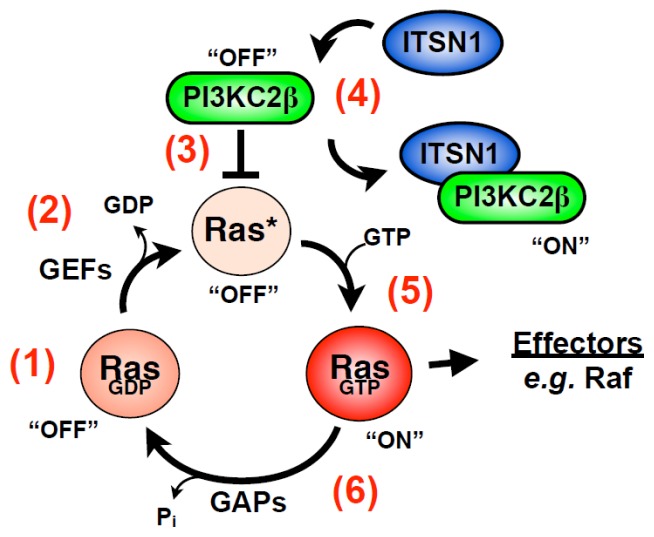
Role of nucleotide-free (nf) Ras in cell signaling. (**1**) RasGDP binds GEFs such as Sos (**2**) which stimulate nucleotide release. Wong *et al*. propose that PI3KC2β traps nf-Ras (**3**) and prevents GTP loading. This interaction inhibits PI3KC2β lipid kinase activity as well as activation of Ras. ITSN1 binding (**4**) to the PRD of PI3KC2β results in Ras dissociation and immediate binding of GTP (**5**) to activate Ras. Dissociation of Ras also activates PI3KC2β. This model suggests the possibility of additional cellular targets of nf-Ras that may also be negatively regulated by the nucleotide-free form and thus activated upon GTP-loading of Ras. These targets, along with PI3KC2β, may contribute to Ras transformation in the absence of binding active RasGTP.

Ras proto-oncogenes (H-Ras, K-Ras and N-Ras) play a central role in human tumorigenesis with greater than 30% of human tumors bearing activating mutations in one of the three Ras genes [97]. However, the role of Ras in tumorigenesis is complex. In some contexts, Ras exhibits tumor suppressor activity. For example, skin and lung tumors with constitutively active Ras alleles exhibit loss of the wild type allele [98,99]. Indeed, wild type Ras suppresses the tumorigenic properties of mutated oncogenic Ras in both *in vitro* and *in vivo* models of tumorigenesis [100–107]. The mechanism behind this phenomenon is unclear but may involve interaction between mutated Ras and its wild type counterpart. In addition, the tumor response may depend on the isoforms of Ras interacting (H-, K-, or N-Ras) as well as the type of tumor [104,108]. The discovery that nucleotide-free Ras inhibits specific signaling pathways provides another potential explanation for the antagonistic role of wild type Ras on tumorigenesis [58]. Wong and colleagues demonstrated that nucleotide-free binds and inhibits PI3KC2β lipid kinase activity *in vitro*. They proposed that in the absence of Ras mutations, nucleotide-free Ras negatively regulates specific biochemical targets (Figure 2). Such interaction has at least two predicted consequences: inhibition of the Ras binding protein (e.g., PI3KC2β) and inhibition of Ras loading with GTP. Mutational activation of one allele of Ras coupled with subsequent loss of the remaining wild-type allele would thus lead to loss of this repression and subsequent activation of these pathways. This model predicts that there may be targets of Ras important for tumorigenesis that does not bind active Ras. As discussed above, PI3KC2β is beginning to emerge as one such target.

### 6.2. ITSN and Asymmetric Cell Division: Maintenance of Cancer Stem Cells

Cell division does not always give rise to identical cells, as cellular components are frequently distributed asymmetrically between two daughter cells. This process is necessary for partitioning of important proteins for patterning and differentiation [109,110]. Asymmetric division is also a characteristic of stem cells important for maintenance of self-renewal potential. One of the forces leading to asymmetric cell division is the presence of a polar environment, which is a well-known event regulating development. Cells have intrinsic determinants that drive asymmetric division. One of the most important and conserved determinants of cell fate is Numb. In dendritic spines, ITSN1 binds Numb, an adaptor protein important for distribution of Notch during division [67] as well as for inhibition of Notch activity [109]. Numb is also an activator of PARs, which are components of the polarity complex. Asymmetric cell division is also regulated by the mitotic spindle, which defines cell size during division. This event is regulated by the polarity complex composed of PAR proteins and atypical PKC (aPKC). Dap160 regulates the PAR3-PAR6-atypical PKC polarity complex in *Drosophila* neuroblasts [69]. Dap160 binds aPKC to enhance its kinase activity, and Dap160 interacts with PAR6 to block its inhibition of aPKC [69]. Dap160 overexpression leads to asymmetric distribution of aPKC in neuroblasts increasing the number of neuroblasts that proliferate [69]. The role of ITSN-aPKC in mammals has not been explored. However, ITSN1-Numb interactions suggest that ITSNs may play a pivotal role in regulation of asymmetric division [67].

### 6.3. ITSN’s and Regulation of Receptor Trafficking: Implications for Cancer

The regulation of receptor trafficking plays an important role in cancer. As mentioned above, ITSN1 regulates receptor trafficking (reviewed in [13]). ITSN1 interacts with several endocytic proteins such as clathrin, AP2, Eps15 and dynamin (as reviewed in [13,21]). Like Eps15, ITSN1’s EH domains may play a role in regulating receptor internalization. Teckchandani and colleagues demonstrate that Eps15 and ITSNs regulate integrin β1 internalization through the Dab2 adaptor protein [111]. Integrin β1 appears to be a Dab2-specific cargo as mutations in the EH-binding sites of Dab2 impair integrin β1 endocytosis without affecting transferrin internalization. Given the observation that ITSN1 regulates integrin β1 phosphorylation, these data suggest that ITSN1 may regulate integrin β1 internalization through multiple pathways. ITSNs may regulate trafficking of other receptors as well as suggested by their ability to interact with the Rab and Arf GTPase pathways [45]. Both Arf6 and Rab11 are important for receptor recycling [112]. Thus, ITSNs may play a broad role in regulating many membrane bound receptors through these processes of internalization and recycling. In addition, recycling is important for localization of integrin β1 to different cellular compartments. The Eps15 homology domain 1 (EHD1) protein binds Rab11-FIP2, a target of Rab11, and regulates receptor recycling [113]. Rab11-FIP2 was identified as an ITSN-binding protein [45], and thus this ITSN-Rab11-FIP2 complex may contribute to oncogenesis through regulation of membrane receptor recycling. The recycling machinery also regulates E-cadherin levels [114,115]. The balance of recycling *versus* degradation of E-cadherin plays an important role in oncogenic transformation [116]. Finally, ITSN1 regulates exocytosis through interaction with members of the Q-SNARE family such as SNAP-23/25, which are important for docking vesicles at the plasma membrane and for secretory granule exocytosis [30,36]. However, only ITSN1-L has been directly linked to regulation of exocytosis through its Cdc42 GEF activity [3,117] even though ITSN1-S also interacts with SNAP-25.

Ding and colleagues identified human ITSN2 as a novel Eps8-interacting protein by yeast two-hybrid screening [118]. Eps8 plays an important role in EGFR-dependent signaling and malignant transformation. Eps8 bound ITSN2’s CC region, and ITSN2 overexpression induced the degradation of Eps8 by a lysosomal-mediated pathway. Therefore, ITSN2 may act as a tumor suppressor. Whether this involves modulation of Eps8 ubiquitylation to target the protein to the lysosome remains to be determined. However, their results indicate that ITSN2-induced degradation of Eps8 is not mediated by the proteasome.

## 7. Future Directions

The past 15 years have witnessed an increasing interest in the ITSN family of scaffolds. While these multi-domain scaffolds play critical roles in regulating the endocytic pathway, it is becoming apparent that their function is not restricted to this single biochemical process. Rather, these scaffolds play a more complex role in cell biology, regulating multiple biochemical pathways important for both normal and pathophysiological processes. It will be critical for future studies to delineate the specific mechanisms by which ITSNs participate in these pathways and the biological importance of this regulation.

## Figures and Tables

**Table 1:** ITSN orthologs.

Species 1	# genes 2	ITSN1	ITSN2	GEF domain 3
*Homo sapiens*	2	Chr 21	Chr 2	Yes
*Pan troglodytes*	2	Chr 21	Chr 2A	Yes
*Macaca mulatta*	2	Chr 19	Chr 2	Yes
*Callithrix jacchus*	2	Chr 21	Chr 14	Yes
*Nomascus leucogenys*	2	ND 5	ND	Yes
*Pongo abelii*	2	Chr 21	Chr 2A	Yes
*Canis lupus familiaris*	2	Chr 31	Chr 17	Yes
*Felis catus*^4^	2	ND	ND	Yes
*Bos taurus*	2	Chr 1	Chr 11	Yes
*Cavia porcellus*	2	ND	ND	Yes
*Mus musculus*	2	Chr 16	Chr 12	Yes
*Rattus norvegicus*	2	Chr 11	Chr 6	Yes
*Cricetulus griseus*	2	ND	ND	Yes
*Ailuropoda melanoleuca*	2	ND	ND	Yes
*Equus caballus*	2	Chr 15	Chr 26	Yes
*Sus scrofa*	2	Chr 13	Chr 3	Yes
*Sorex araneus*	2	ND	ND	Yes
*Tupaia belangeri*^4^	2	ND	ND	Yes
*Oryctolagus cuniculus*	2	ND	ND	Yes
*Loxodonta africana*^4^	2	ND	ND	Yes
*Monodelphis domestica*	1	Chr 4	-	Yes
*Erinaceus europaeus*^4^	1	ND	ND	? 6
*Echinops telfairi*^4^	2	ND	ND	Yes
*Anolis carolinensis*	2	Chr 3	Chr 1	Yes
*Meleagris gallopavo*	2	Chr1	Chr 2	Yes
*Gallus gallus*	2	Chr 1	Chr 3	Yes
*Myotis lucifugus*^4^	1	-	ND	Yes
*Xenopus laevis*	2	ND	ND	Yes 9
*Xenopus tropicalis*	2	ND	ND	Yes
*Ornithorhynchus anatinus*	2	ND	Chr 2	Yes
*Oryzias latipes*	2	Chr 24	Chr 3	Yes
*Oreochromis niloticus*	2	ND	ND	Yes
*Danio rerio*^7^	3	Chr 1	Chr 20/Chr 17	Yes
*Takifugu rubripes*^7^	3	ND	ND/ND	Yes
*Tetraodon nigroviridis*^7^	3	Chr 17	Chr 5/Chr 14	Yes
*Taeniopygia guttata*	2	Chr 1B	Chr 3	Yes
*Gasterosteus aculeatus*^7^	3	Group VI	Group II & Group XVIII	Yes
*Ciona savignyi*	1	ND	-	No
*Ciona intestinalis*	2	ND	ND	Yes
*Nasonia vitripennis*	1	ND	-	Yes
*Acyrthosiphon pisum*	1	-	ND	Yes
*Anopheles gambiae*^8^	1	3R	-	No
*Aedes aegypti*^8^	1	ND		No
*Drosophila pseudoobscura*^8^	1	ND	-	No
*Drosophila melanogaster*^8^	1	2L	-	No
*Tribolium castaneum*^4^	1	LG3	-	? 6
*Apis mellifera*^8^	1	-	ND	Yes
*Caenorhabditis briggsae*	1	ND	-	No
*Caenorhabditis elegans*	1	Chr 4	-	No
*Caenorhabditis remanei*	1	ND	-	No
*Hydra magnipapillata*	2	ND	ND	Yes
*Strongylocentrotus purpuratus*	2	ND	ND	? 6
*Cryptococcus neoformans*	1	ND	-	Yes

1 Protein sequences were obtained from NCBI, and information was derived by searching NCBI MapView (http://www.ncbi.nlm.nih.gov/projects/mapview/) for intersectin related sequence, and the SMART database (http://smart.embl-heidelberg.de) for entries possessing EH, SH3, and DH–PH domains. Additional comparisons were done by examining various ITSN-related proteins using BLAST programs to determine relationships between orthologs;

2 Denotes the number of distinct genetic loci encoding ITSN-related proteins in the indicated species. NOTE: many species also contain one or more ITSN pseudogenes;

3 Indicates whether the *ITSN* genes encode a long splice variant encoding a Cdc42 GEF;

4 Due to ambiguous sequence, protein appears to have less than 5 SH3 domains;

5 ND, not determined;

6 Partial clones;

7 These species possess three *ITSN* genes. The increase in *ITSN* genes likely arose from a genome duplication event in vertebrate evolution [19];

8 These orthologs possess 4 SH3 domains in contrast to the 5 SH3 domains found in most ITSN orthologs;

9 Described in [20].
